# Oxidative Stress during the Progression of *β*-Amyloid Pathology in the Neocortex of the Tg2576 Mouse Model of Alzheimer's Disease

**DOI:** 10.1155/2015/967203

**Published:** 2015-04-20

**Authors:** Sara Porcellotti, Francesca Fanelli, Anna Fracassi, Sara Sepe, Francesco Cecconi, Cinzia Bernardi, AnnaMaria Cimini, Maria Paola Cerù, Sandra Moreno

**Affiliations:** ^1^Department of Science, LIME, University Roma Tre, Viale Guglielmo Marconi, No. 446, 00146 Rome, Italy; ^2^IFOM Foundation, FIRC Institute of Molecular Oncology Foundation, Via Adamello 16, 20139 Milan, Italy; ^3^IRCCS Santa Lucia Foundation, Via del Fosso di Fiorano 64, 00143 Rome, Italy; ^4^Department of Radiological Sciences and Laboratory Medicine, UOC Pathological Anatomy, San Filippo Neri Hospital, Via Giovanni Martinotti 20, 00135 Rome, Italy; ^5^Department of Life, Health and Environmental Sciences, University of L'Aquila, Piazzale Salvatore Tommasi 1, 67100 Coppito, Italy

## Abstract

Alzheimer's disease (AD) is the most common form of dementia, characterized by progressive neurodegeneration. Pathogenetic mechanisms, triggered by *β*-amyloid (A*β*) accumulation, include oxidative stress, derived from energy homeostasis deregulation and involving mitochondria and peroxisomes. We here addressed the oxidative stress status and the elicited cellular response at the onset and during the progression of A*β* pathology, studying the neocortex of Tg2576 model of AD. Age-dependent changes of oxidative damage markers, antioxidant enzymes, and related transcription factors were analysed in relation to the distribution of A*β* peptide and oligomers, by a combined molecular/morphological approach. Nucleic acid oxidative damage, accompanied by defective antioxidant defences, and decreased PGC1*α* expression are already detected in 3-month-old Tg2576 neurons. Conversely, PPAR*α* is increased in these cells, with its cytoplasmic localization suggesting nongenomic, anti-inflammatory actions. At 6 months, when intracellular A*β* accumulates, PMP70 is downregulated, indicating impairment of fatty acids peroxisomal translocation and their consequent harmful accumulation. In 9-month-old Tg2576 neocortex, A*β* oligomers and acrolein deposition correlate with GFAP, GPX1, and PMP70 increases, supporting a compensatory response, involving astroglial peroxisomes. At severe pathological stages, when senile plaques disrupt cortical cytoarchitecture, antioxidant capacity is gradually lost. Overall, our data suggest early therapeutic intervention in AD, also targeting peroxisomes.

## 1. Introduction

Alzheimer's disease (AD) is the most common form of dementia, characterized by progressive neurodegeneration, particularly affecting cortical and hippocampal brain regions [1]. Histopathological features of AD are senile plaques, composed of *β*-amyloid (A*β*) peptide polymers, and intracellular neurofibrillary tangles, formed by hyperphosphorylated Tau protein. A*β* peptide is produced by neurons through the processing of APP and has the propensity to aggregate in oligomers, which are more neurotoxic than A*β* plaques themselves [2].

Two forms of the disease are classified, according to the age of manifestation of the disease, namely, early onset AD (EOAD) and late onset AD (LOAD). The former has been associated with the presence of mutations to the genes encoding for amyloid precursor protein (APP), presenilin 1 and presenilin 2 (PSEN1 and PSEN2) [3]. Based on these genetic studies, several animal models have been so far generated [4], among which the Tg2576 mouse strain harboring the human APPswe mutant allele linked to familial Alzheimer's disease, is one of the most widely used [5]. Although these mice lack neurofibrillary tangles and substantial neuronal loss, they develop early synaptic deficits and late neuropathological features, including amyloid plaques and dystrophic neurites [6]. Therefore, accumulation of A*β* peptide, leading to A*β* pathology, is responsible for age-related memory decline in this model [7, 8], thus reproducing rather faithfully the slow progression of human clinical symptoms and allowing dissecting the mechanisms underlying the onset and progression of disease [6–11].

Growing evidence has demonstrated that oxidative stress is an important factor contributing to the initiation and progression of AD [12]. Reactive oxygen species (ROS) production in AD derives from deregulation of energy homeostasis, hence involving mitochondria and peroxisomes, cellular compartments importantly participating in ROS and lipid metabolism [13, 14].

Imbalance in the redox status activates an array of cell response mechanisms, including expression and activation of nuclear receptors and their cofactors regulating energy metabolism and antioxidant defences [15]. Among these, peroxisome proliferator activated receptors (PPARs), comprising three isotypes (*α*, *β*, and *γ*), regulate both mitochondrial and peroxisomal biogenesis and functions [16, 17]. Importantly, beneficial effects of PPAR agonists on AD-related neurodegeneration are reported by several groups [18, 19], strongly suggesting that therapeutic strategies targeting these molecules are promising in preventing AD onset and progression. Among PPAR coactivators, PGC1*α* is the most extensively studied, for its wide array of functions, including glucose metabolism, fatty acid oxidation, ROS scavenging, and mitochondrial/peroxisomal biogenesis and functioning [20, 21].

In previous works, we reported variations of antioxidant and lipid metabolizing enzymes at the onset [22] and during the progression [10] of A*β* pathology, in Tg2576 (Tg) mice. The former study focused on early changes in neocortical and hippocampal tissues; the latter emphasized the age-dependent role of peroxisomes and PPAR*α* in the hippocampus.

The present work extends the analysis of oxidative stress and the elicited cellular response to the neocortex of Tg mice, aging 3, 6, 9, 12, and 18 months, as compared to wild type (WT) littermates. The variations of oxidative damage markers, redox status sensors, and antioxidant and peroxisomal proteins were studied in relation to the accumulation of neurotoxic A*β* peptide and oligomers.

## 2. Materials and Methods

### 2.1. Animals

Heterozygous female Tg2576 mice [5] and WT littermates were used for all experiments. Male mice (C57B6 × SJL), hemizygous for human APP695 carrying the double mutation K670N and M671L (FADSwedish mutation), were purchased from Taconic Farms, Inc. (Germantown, NY, USA). The Tg colony is maintained and the genotyping is performed as previously described [22]. For all analyses, Tg and WT female mice of 3, 6, 9, 12, and 18 months of age were used.

Experiments were performed in accordance with the European Community's Council Directive 86/609/EEC. Formal approval of these experiments was obtained from the Italian Ministry of Health (D.L.vo 116/92; Prot. number 155-VI-1.1). All efforts were made to minimize the number of animals used and their suffering.

### 2.2. Molecular Analyses

Six animals from each age group (three Tg and three WT) were killed by cervical dislocation, brains were rapidly excised on an ice-cold plate, and cortices were dissected out.

*Cortical Tissue Homogenate Preparation and Protein Extraction.* A lysis buffer (320 mM sucrose, 50 mM NaCl, 50 mM Tris–HCl, pH 7.5, 1% Triton X-100, 0.5 mM sodium orthovanadate, 5 mM *β*-glycerophosphate, and 1% protease inhibitor) was used to homogenate cortical tissue, incubating the samples on ice for 30 min. Homogenates were centrifuged at 13,000 rpm for 10 min at 4°C. The total protein content of the resulting supernatant was determined using a spectrophotometric assay, according to the method described by Bradford [23]. Samples were then diluted 3 : 4 in 200 mM Tris–HCl, pH 6.8, containing 40% glycerol, 20% *β*-mercaptoethanol, 4% sodium dodecyl sulphate (SDS), and bromophenol blue.

### 2.3. Western Blotting (WB)

In WB experiments, performed as previously described [22], membranes were probed at 4°C overnight with either of the following primary antibodies: 1 : 200 rabbit polyclonal antiglial fibrillary acidic protein (anti-GFAP, Sigma-Aldrich, St. Louis, MO, USA); 1 : 1000 rabbit polyclonal anti-SOD1 (Abcam, Cambridge Science Park, Cambridge, UK); 1 : 10000 anti-SOD2 (Abcam); 1 : 3000 rabbit polyclonal anti-GPX1 (Abnova, Taipei City, Taiwan); 1 : 2000 rabbit polyclonal anti-CAT (Rockland, Gilbertsville, PA, USA); 1 : 1000 rabbit polyclonal anti-PMP70 (Sigma-Aldrich); 1 : 10000 rabbit polyclonal anti-Pex14p (generous gift from Professor R. Erdmann, Ruhr University Bochum, Bochum, Germany); 1 : 1000 rabbit polyclonal anti-AOX (generous gift from Professor A. Völkl, University of Heidelberg, Heidelberg, Germany); 1 : 1000 rabbit polyclonal anti-THL (generous gift of Professor P. Van Veldhoven, Katholieke Universiteit Leuven, Leuven, Belgium); and 1 : 3000 mouse monoclonal anti-*β*-actin (Sigma-Aldrich). Membranes were then incubated with 1 : 2000 HRP-conjugated goat anti-rabbit or anti-mouse IgG secondary antibodies (Santa Cruz Biotechnology, Santa Cruz, CA, USA) in blocking solution, for 1 h at 4°C. Immunoreactive bands were visualized by a chemiluminescence detection kit (ECL Plus Western Blotting Detection Reagents, Amersham GE Healthcare, Little Chalfont, UK). The relative densities of immunoreactivity were determined by densitometry using the software ImageJ (NIH, Bethesda, MD, USA) and normalized with respect to *β*-actin. Data are mean of five different experiments.

### 2.4. Statistical Analyses

Statistical evaluation of WB experiments was performed using GraphPad Prism 4 software. For comparison between genotypes and among different ages, statistical analysis of WB densitometric values was accomplished using two-way analysis of variance (ANOVA) followed by the Bonferroni test to detect significant differences between groups. Means from independent experiments were then expressed as means ± SD. For all statistical analyses, *P* < 0.05 was considered as statistically significant.

### 2.5. Morphological Analyses

#### 2.5.1. Brain Tissue Preparation and Sectioning for Light and Confocal Microscopy

For each age considered, 3 Tg mice and 3 WT littermates were deeply anesthetized with urethane (1 g/kg body weight, injected i.p.), before rapid killing by transcardial perfusion with the fixative solution, as previously described [22]. Brains were dissected out, sagittally cut along the midline, and paraffin-embedded.

Serial, 5 *μ*m thick sagittal sections from each Tg and WT brain sample were obtained by a rotary microtome, till exhaustion of the specimens. For each marker to be analyzed, five sections from each brain sample were processed.

#### 2.5.2. Congo Red Staining

Deparaffinized sections were treated with hematoxylin for 15 minutes, immersed in a 0.5% acid ethanol solution and then in a Congo Red solution (composed of 2.5% NaCl, 50% ethanol solution, 0.01% KOH solution, and 0.2% Congo Red). Finally, sections were dehydrated and mounted with Eukitt. Slides were observed in an Olympus BX 51 microscope, under polarized light, to detect amyloid plaques for their birefringence. All the stained sections were extensively analysed and representative electronic images were captured by a Leica DFC 420 camera, using a Leica Application Suite system, and composed in an Adobe Photoshop CS5 format.

#### 2.5.3. Immunohistochemistry (IHC)

For light microscopic immunolocalization, deparaffinized sections were processed as previously described [22]. The following primary antibodies were used: 1 : 100 mouse monoclonal anti-NeuN (Chemicon, Temecula, CA, USA); 1 : 300 rabbit polyclonal anti-GFAP (Dako, Glostrup, Denmark); 1 : 100 mouse monoclonal anti-A*β* (Synaptic Systems, Gottingen, Germany); 1 : 100 mouse monoclonal anti-oligo-pGlu A*β* (Synaptic Systems); 1 : 100 mouse monoclonal anti-Iba1 (Abcam); 1 : 500 mouse monoclonal anti-8-hydroxy(deoxy)guanosine (8-OHG) (QED Bioscience Inc., San Diego, CA, USA); 1 : 500 rabbit polyclonal anti-acrolein (generous gift from Professor K. Uchida, Nagoya University, Nagoya, Japan); 1 : 200 rabbit polyclonal anti-PPAR*α* (ABR Affinity BioReagents, Golden, CO, USA); 1 : 50 mouse monoclonal anti-PGC1*α* (Calbiochem, Darmstadt, Germany); 1 : 100 rabbit polyclonal anti-SOD1 and 1 : 1000 anti-SOD2; 1 : 100 rabbit polyclonal anti-GPX1; and 1 : 200 rabbit polyclonal anti-PMP70. In control sections, the primary antibody was omitted. Biotinylated goat anti-rabbit or anti-mouse IgG (Vector Laboratories, Burlingame, CA, USA) were used as secondary antibodies and immunocomplexes were visualized, as previously reported [22]. Slides were dehydrated and mounted and then observed under an Olympus BX 51 microscope, equipped with a Leica DFC 420 camera. All immunoreacted sections were extensively analysed and representative electronic images were captured by a Leica Application Suite system and composed in an Adobe Photoshop CS5 format.

#### 2.5.4. Immunofluorescence (IF)

For double immunofluorescence, deparaffinized sections from 3-month-old Tg brains were incubated with a mixture of 1 : 1000 anti-SOD2 and 1 : 500 anti-8-OHG, overnight at 4°C. Slides were thoroughly rinsed with PBS and then incubated for 1 h at RT with a mixture of 1 : 500 Chromeo 488-conjugated goat anti-rabbit IgG and 1 : 500 Chromeo 555-conjugated goat anti-mouse IgG (both from Invitrogen, Life Technologies Italia, Monza, Italy). All antibodies were diluted in PBS containing 4% BSA. Controls were performed by omitting the primary antibodies. Slides were mounted with Vectashield (Vector) and observed in a Leica TCS SP5 confocal microscope. Representative images, captured by a Leica Application Suite system, were composed in an Adobe Photoshop CS5 format.

#### 2.5.5. Electron Microscopy

For the ages of 3, 6, and 9 months, one Tg mouse and one WT littermate were anesthetized and perfused, as described for light microscopy. Brains were dissected out and sagittally cut by a vibratome, obtaining 100 *μ*m thick sections, to be processed for ultrastructural studies. Osmium postfixation, dehydration, and Epon flat-embedding were performed as previously described [22]. Select neocortical fields of resin-embedded sections, identified in a light microscope, were remounted on Epon blanks and further sectioned on a Reichert Ultracut S ultramicrotome. Ultrathin sections were briefly contrasted with uranyl acetate and observed in a Philips CM120 electron microscope equipped with a Philips Megaview III camera. Representative images were captured by AnalySys 2.0 software and composed in Adobe Photoshop CS5 format.

## 3. Results

### 3.1. Neural Cell Markers and Amyloid Deposits

**Figure 1:**
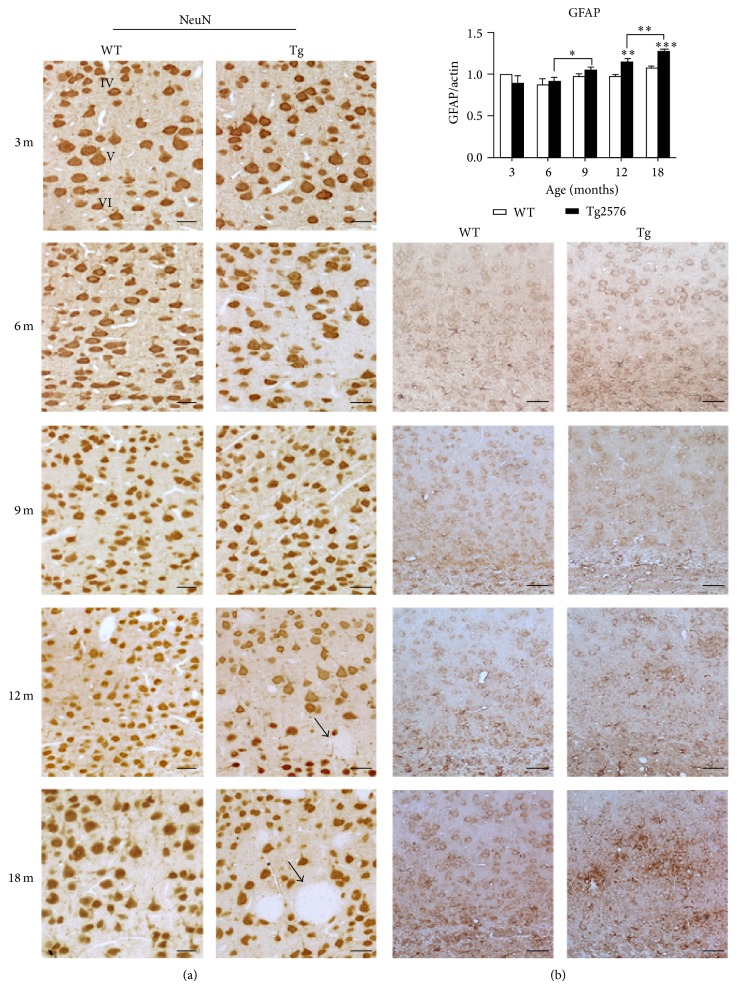
NeuN and GFAP expression in the neocortex of WT and Tg mice. (a) NeuN IHC localization in the neocortex of 3-, 6-, 9-, 12-, and 18-month-old WT and Tg mice. Arrows indicate amyloid plaques in 12- and 18-month-old mice. Scale bars, 40 *μ*m. (b)* Upper picture*, GFAP WB densitometric analysis on neocortex protein extracts of 3-, 6-, 9-, 12-, and 18-month-old mice. Values are expressed as mean ± SD. ^∗∗^ *P* < 0.01; ^∗∗∗^ *P* < 0.001. (b)* Lower pictures*, GFAP IHC distribution in 6-, 9-, 12-, and 18-month-old WT and Tg neocortex. Scale bars, 60 *μ*m.

To analyse cortical cytoarchitecture, the localization of the neuronal marker NeuN and the astroglial marker GFAP was studied by IHC (Figure 1).

**Figure 2:**
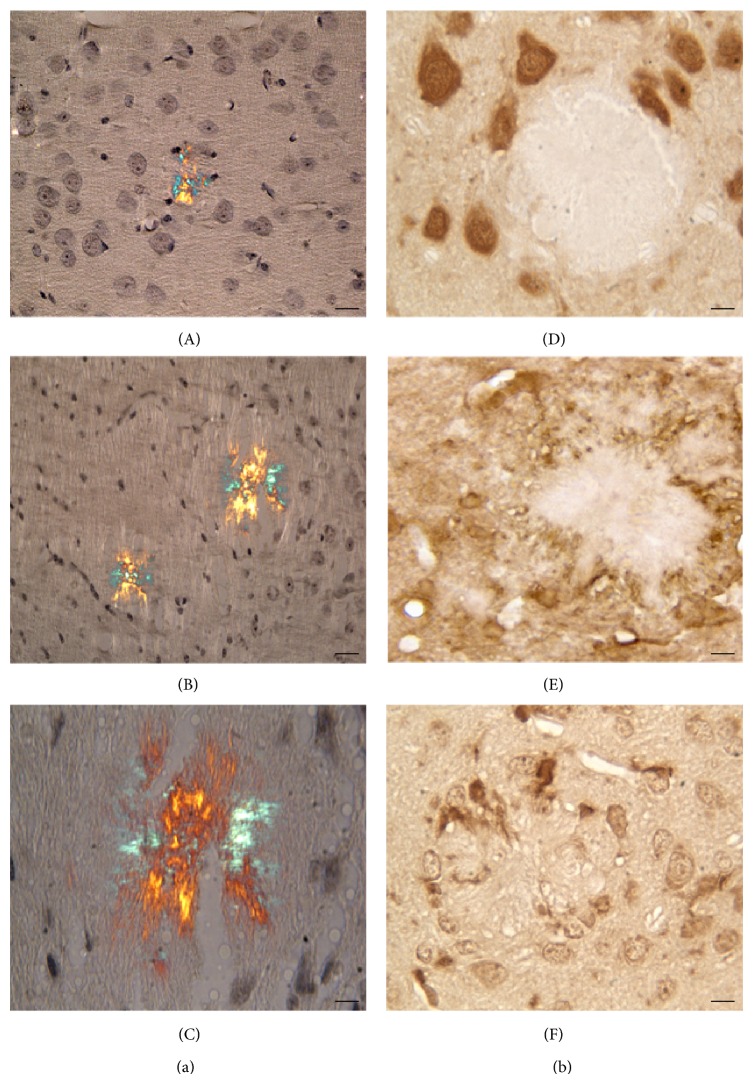
Senile plaques in Tg neocortex. (a) Congo Red staining, observed under polarized light. Small, birefringent structures are first detected at 9 months (A), while larger plaques are seen at 18 months ((B) and (C)). Scale bars A and B, 30 *μ*m; C, 15 *μ*m. (b) Neocortical areas of 18-month-old Tg mice, showing large senile plaques, after IHC for NeuN (D), GFAP (E), and Iba1 (F). Glial cells, but not neuronal cell bodies, are seen within the aggregates. Scale bars, 15 *μ*m.

Alterations in the distribution of NeuN positive cells are only observed in Tg at late stages (12 and 18 months), when the overall layering is disrupted by the presence of large and progressively more numerous amyloid plaques. These deposits, found throughout the layers of the neocortex, are totally devoid of NeuN positive cells (Figures 1 and 2).

Expression of GFAP, revealed by IHC and WB, is unchanged until 9 months, while a sustained increase in immunopositivity in Tg neocortex is detected thereafter (Figure 1). At 18 months of age, numerous hypertrophic astrocytes are found throughout the layers and, particularly, around senile plaques (Figures 1 and 2). Similar localization is shown by Iba1-positive microglia (Figure 2).

Senile plaques were recognised by Congo Red staining, using a polarizing filter (Figure 2). Small birefringent amyloid deposits are first detected in 9-month-old Tg neocortex. Their size and number progressively increase during aging, leading to cortical cytoarchitecture derangement.

**Figure 3:**
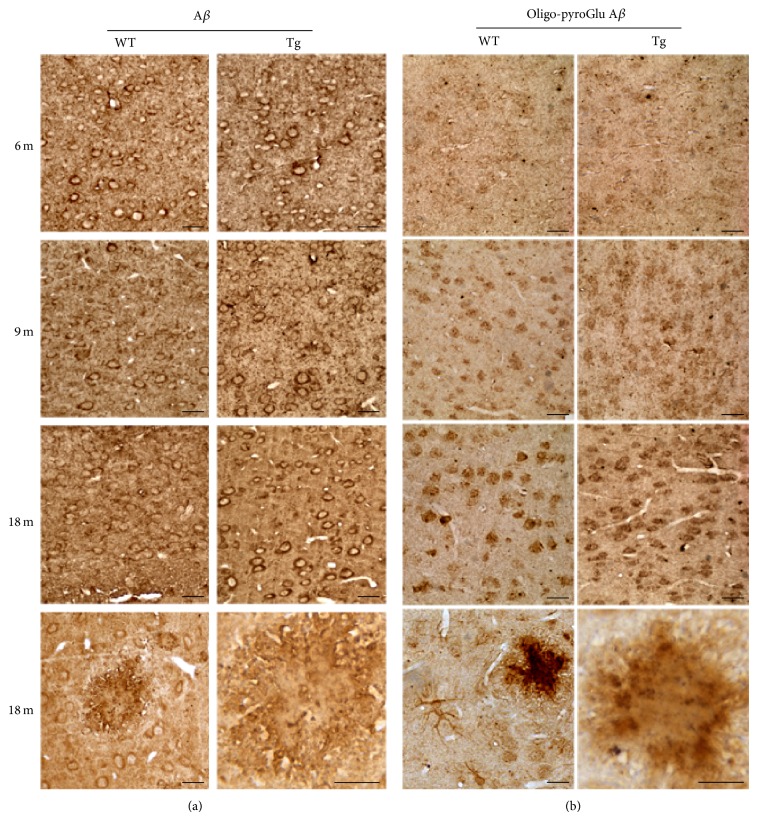
Amyloid deposits in WT and Tg neocortex. (a) A*β* IHC in 6-, 9-, and 18-month-old WT and Tg neocortex. A stronger staining is consistently present in Tg neurons, compared to WT. At late stages, A*β* accumulates within senile plaques. Scale bars, 40 *μ*m. (b) Immunolocalization of anti-oligo-pyroGlu A*β*, revealing low molecular weight oligomers. The reaction product accumulates in Tg neocortex starting from 9 months and is observed both intra- and extracellularly, particularly inside the plaques. Scale bars, 40 *μ*m.

Since A*β*, particularly in its oligomeric form, is considered the primary culprit of AD pathology [2], we studied the distribution of A*β* peptides and respective oligomers by IHC (Figure 3). The antibody to A*β*, recognising 1–38, 1–40, and 1–42 A*β* peptides, reveals no difference between WT and Tg at 3 months (not shown). A stronger immunoreactivity of Tg neurons, compared to WT, is instead observed starting from 6 months of age (Figure 3(a)). At later stages, A*β* accumulates extracellularly, especially within senile plaques.

Low molecular weight oligomers were immunolocalized by anti-oligo-pGlu A*β*, a truncated and pyroglutamate-modified form of A*β*, composing the central nucleus of plaques on which all other native A*β*s aggregate [24]. Immunostaining accumulates in Tg neocortex starting from 9 months of age and is observed both intra- and extracellularly, particularly inside the plaques (Figure 3(b)).

### 3.2. Ultrastructural Analysis

**Figure 4:**
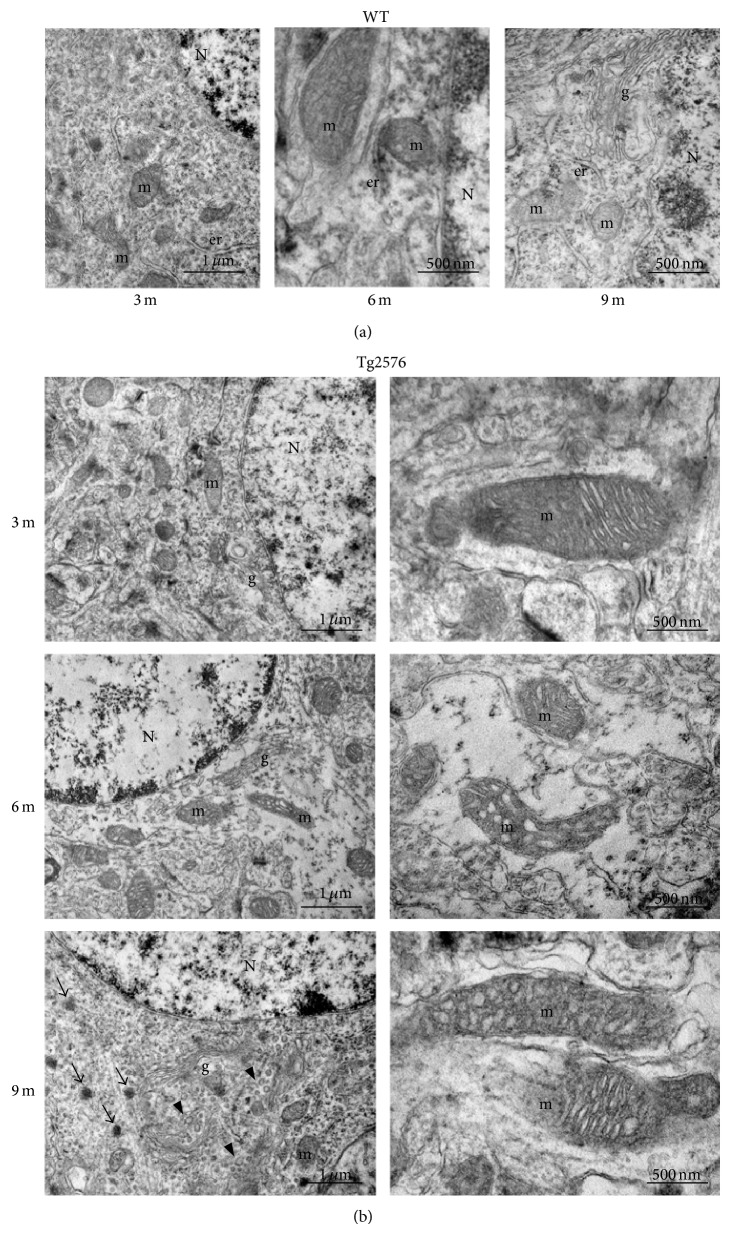
Ultrastructural analyses of WT and Tg neocortex. (a) WT neocortical neurons from 3-, 6-, and 9-month-old animals. Regular ultrastructural features of the nuclear compartment (N) and cytoplasmic organelles (m, mitochondrion; er, endoplasmic reticulum; g, Golgi complex) are observed at all ages. (b) Tg neocortical neurons showing progressive damage, starting from 6 months of age. Mitochondrial (m) abnormalities include outer and inner membrane derangement, with disrupted* cristae* and vacuolization. Abnormally dilated Golgi complex (g),* cisternae* fragmentation (arrowheads), and numerous dense bodies (arrows) are also readily identified in 9-month-old Tg neocortical neurons.

Ultrastructural analysis performed on the neocortex of 3-, 6-, and 9-month-old animals shows genotype- and age-related differences (Figure 4). While 3-month-old samples, irrespective of the genotype, show normal ultrastructural features in both nuclear and cytoplasmic compartments, at 6 and 9 months this morphology is only conserved in WT neocortex (Figure 4(a)). By contrast, mild-to-severe lesions are detected in Tg neurons as a function of age, starting from 6 months (Figure 4(b)). Mitochondria are probably the most profoundly affected organelles, in that their outer and inner membranes appear irregularly arranged, with disrupted* cristae* and vacuolization. Abnormally dilated Golgi complex, accompanied by* cisternae* fragmentation, is also readily identified in Tg cells. Interestingly, in 9-month-old Tg neocortex, the occurrence of dense bodies is a frequent finding.

### 3.3. Oxidative Stress Markers: 8-OHG and Acrolein

**Figure 5:**
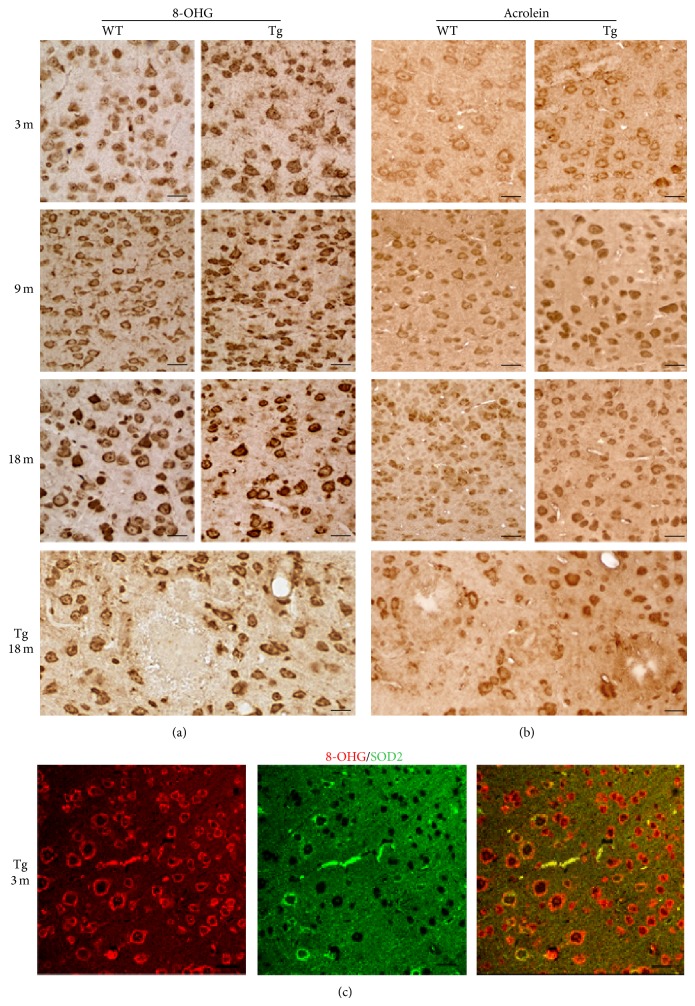
Expression of oxidative damage markers in WT and Tg neocortex. (a) 8-OHG IHC in 3-, 9-, and 18-month-old WT and Tg neocortex. Prominent cytoplasmic staining in Tg neurons is observed throughout the ages. Scale bars, 30 *μ*m. (b) Acrolein IHC showing higher levels in Tg than in WT neurons, starting from the age of 9 months. Scale bars, 30 *μ*m. (c) Double immunofluorescence of 8-OHG (red) in combination with SOD2 (green) in the neocortex of 3-month-old Tg mice, showing partial colocalization of both markers, thus indicating mitochondrial DNA oxidative modifications. Scale bars, 30 *μ*m.

In view of the central role played by oxidative stress in AD and of the specific involvement of A*β* in ROS generation, we searched for oxidatively modified macromolecules. To this aim, the occurrence of 8-OHG and acrolein, as markers of oxidative damage to nucleic acids and lipids, respectively, was investigated by IHC (Figure 5).

Age- and genotype-dependent alterations are detected for both markers. In particular, 8-OHG immunoreactivity levels are consistently higher in Tg, compared to WT, starting from 3 months. At 18 months, Tg neocortical plaques appear surrounded by 8-OHG-immunoreactive neuronal and glial cells, being negative themselves. Immunostaining is mainly localized to the cytoplasm, indicating modifications to mitochondrial nucleic acids and cytosolic RNA. Indeed, double IF in 3-month-old Tg neocortex demonstrates partial colocalization of the mitochondrial marker SOD2 with 8-OHG (Figure 5(c)). Interestingly, neurons expressing the highest levels of the antioxidant enzyme show relatively faint staining for the oxidative damage marker.

Acrolein, the most reactive aldehyde produced by lipid peroxidation, is consistently found in Tg neocortex at higher concentrations than in WT starting from 9 months (Figure 5). Senile plaques appear devoid of acrolein, but positive cells are found in their close proximity.

### 3.4. Transcriptional Regulators: PPAR*α* and PGC1*α*

The expression and distribution of the oxidative stress sensors PPAR*α* and PGC1*α* were investigated by IHC, at the onset and during the progression of A*β* pathology, as compared to normal brain aging.

**Figure 6:**
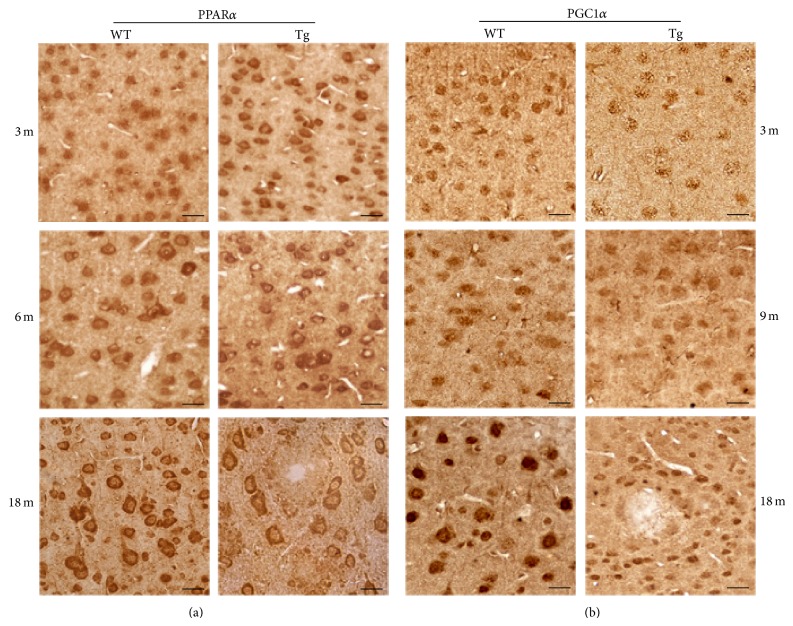
PPAR*α* and PGC1*α* immunolocalization in the neocortex of WT and Tg mice. (a) PPAR*α* IHC in 3-, 6-, and 18-month-old WT and Tg neocortex. Immunoreactivity levels are relatively high in Tg neurons at 3 months and show a mostly cytoplasmic localization. At 18 months, IHC intensity is lower in Tg than in WT neurons. Scale bars, 30 *μ*m. (b) PGC1*α* IHC in 3-, 9-, and 18-month-old WT and Tg neocortex. Consistently low staining levels are observed in Tg neurons. Scale bars, 30 *μ*m.

Extensive analysis of PPAR*α* immunoreacted sections (Figure 6(a)) revealed more intense positivity in Tg neocortex than in WT at 3 months, which however decreased thereafter, becoming fainter than in WT at 18 months. Interestingly, immunostaining, especially high in pyramidal cells (according to [25]), shows a somewhat different localization depending on the age and genotype. While in WT neocortex it is first (3 months) observed in the nucleus, progressively shifting to the cytoplasm, in Tg it appears predominantly cytoplasmic since the earliest stage.

Differently from PPAR*α*, PGC1*α* immunoreactivity at 3 months is lower in Tg than in WT neocortex (Figure 6(b)). Even though age-related increases are detected in both conditions, this genotype-based difference is maintained throughout the examined period and particularly apparent at 18 months. Similarly to PPAR*α*, amyloid plaques are themselves PGC1*α* negative, while being surrounded by immunopositive cells.

### 3.5. Antioxidant Enzymes: SOD1, SOD2, and GPX1

The expression of ROS-scavenging enzymes was studied in WT and Tg neocortex by morphological and biochemical techniques.

**Figure 7:**
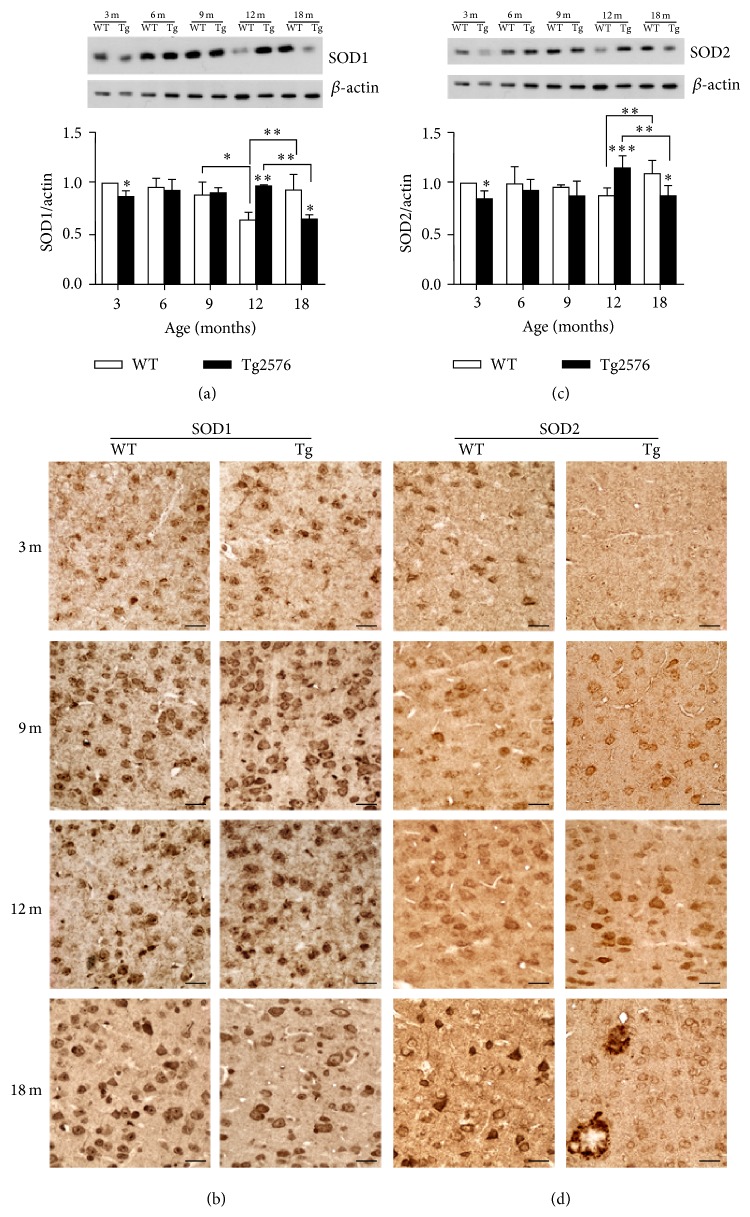
SOD1 and SOD2 protein levels and distribution in WT and Tg neocortex. (a) Densitometric values of SOD1 WB of protein extracts from 3-, 6-, 9-, 12-, and 18-month-old WT and Tg neocortex. Data are expressed as mean ± SD. ^∗^ *P* < 0.05; ^∗∗^ *P* < 0.01. (b) SOD1 IHC in WT and Tg neocortex. Higher expression levels in Tg than in WT neurons are found at 12 months, while the opposite is true for 18 months. Scale bars, 40 *μ*m. (c) Densitometric values of SOD2 WB of protein extracts from 3-, 6-, 9-, 12-, and 18-month-old WT and Tg neocortex. Data are expressed as mean ± SD. ^∗^ *P* < 0.05; ^∗∗^ *P* < 0.01; ^∗∗∗^ *P* < 0.001. (d) SOD2 IHC in WT and Tg neocortex. Relatively low immunoreactivity levels in Tg neurons are found at 3 and 18 months, while at 12 months SOD2 expression is induced in Tg. Note striking immunostaining in glial cells surrounding and infiltrating amyloid plaques. Scale bars, 40 *μ*m.

SOD1 and SOD2 show early (3 months) downregulation in Tg neocortex, as demonstrated by both WB and IHC (Figure 7). No significant genotype-based differences are then detected at 6 and 9 months of age, while, at 12 months, higher expression levels in Tg than in WT neocortex are observed. This pattern reverts in 18-month-old neocortex, when Tg values are lower than WT ones. Concerning SOD2, remarkably strong immunoreactivity is detected in selected cortical neurons and in glial cells surrounding amyloid plaques.

**Figure 8:**
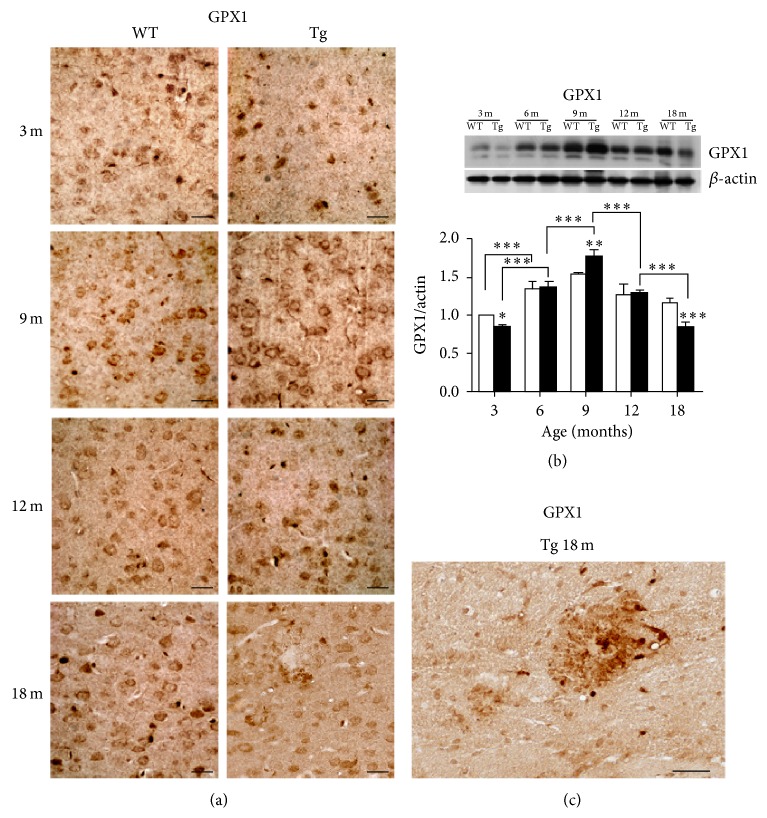
GPX1 expression and distribution in WT and Tg neocortex. GPX1 shows a strongly age- and genotype-related IHC pattern (a) consistent with WB densitometric data (b). Higher expression levels at 9 months and lower levels at 3 and 18 months are detected in Tg neocortex, as compared to WT. Values are expressed as mean ± SD. ^∗^ *P* < 0.05; ^∗∗^ *P* < 0.01; ^∗∗∗^ *P* < 0.001. The higher magnification micrograph (c) shows elevated immunoreactivity in glial cells surrounding and infiltrating an amyloid plaque of 18-month-old Tg neocortex. Scale bars, 40 *μ*m.

In both genotypes, GPX1 shows an age-related pattern characterized by progressive increase of protein levels from youth to maturity, followed by a decrease to minimal values in senescence (Figure 8). This pattern appears exacerbated in Tg neocortex, where the protein is downregulated with respect to WT at 3 months, overexpressed at 9 months, and again downregulated at 18 months, when immunostaining is almost exclusively concentrated at the senile plaques.

### 3.6. Peroxisomal Proteins

**Figure 9:**
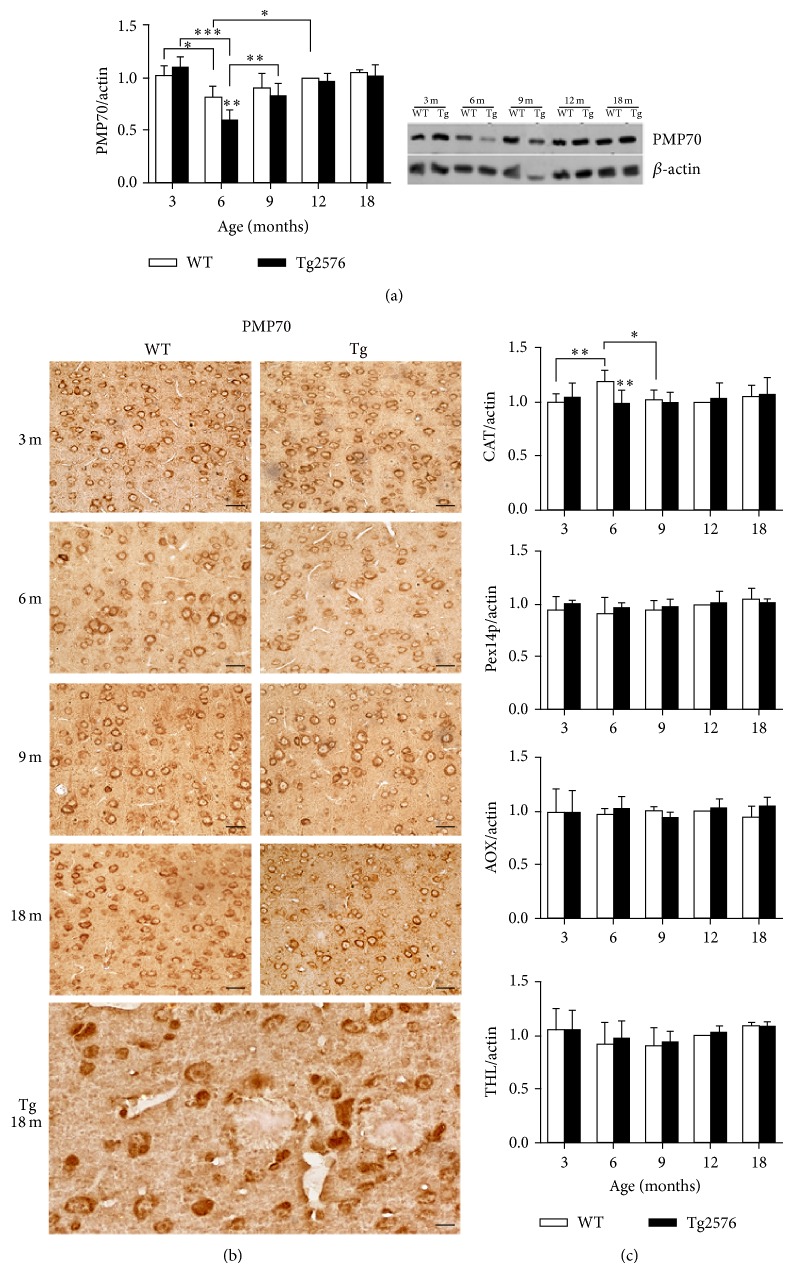
Peroxisomal protein levels and distribution in the neocortex of WT and Tg mice. PMP70 expression showing downregulation at 6 months, as assessed by WB densitometry (a) and IHC distribution (b). Enhancement of PMP70 expression is observed in 9-month-old Tg neurons. Scale bars, 40 *μ*m. (c) Densitometric analyses of WB for CAT, Pex14p, AOX, and THL performed on neocortical protein extracts from 3-, 6-, 9-, 12-, and 18-month-old mice. Significant downregulation of CAT in 6-month-old Tg neocortex is detected. Values are expressed as mean ± SD. ^∗^ *P* < 0.05; ^∗∗^ *P* < 0.01; ^∗∗∗^ *P* < 0.001.

Data obtained by WB and IHC on peroxisomal proteins are reported in Figure 9. While no age- or genotype-based differences of protein levels are detected for CAT, Pex14p, AOX, or THL, significant variations are observed for PMP70. This protein, involved in the transport of long and methyl-branched acyl-CoA esthers [26, 27] across the peroxisomal membrane, is highly expressed at 3 months in both genotypes and significantly downregulated at 6 months, particularly in the Tg neocortex, where the protein is almost reduced by half. A novel increase is observed in 9-month-old Tg, and these levels are maintained thereafter.

## 4. Discussion

The aim of this work was to study age-dependent expression of some key biochemical markers of AD, with an emphasis on oxidative stress and antioxidant enzymatic response. We focused on the neocortex, as one of the most profoundly affected brain regions [1]. As a suitable model for this purpose and consistent with our previous investigations [9, 10, 22, 28], we chose the Tg2576 mouse model [5], which, differently from other models, displays a slowly progressive A*β* pathology. This offers the unique opportunity to study even subtle age-dependent alterations and, more importantly, to identify early biomarkers of the pathology and dissect the molecular mechanisms underlying the first symptoms [9, 22, 29–31]. In view of the greater amyloid load found in females of this specific transgenic model, we chose this gender [32].

In the present work, by comparing Tg animals with their WT littermates, we were able to detect early alterations of pathways related to homeostasis of cellular redox status. Indeed, reduced levels of the antioxidant enzymes GPX1, SOD1, and SOD2 are found in 3-month-old Tg neocortex. Moreover, the bioenergy sensor PGC1*α*, major regulator of their expression [33], shows low immunoreactivity levels in 3-month-old Tg neocortex. The role of this coactivator in AD is emphasized by reports on reduced levels of PGC1*α* in human patients and by the finding that PGC1*α* overexpression in Tg2576 primary neurons decreases A*β* levels [20]. Thus, our observations further support the concept that oxidative stress is a primary culprit in AD pathogenesis [34–36], in overall agreement with our previous study on the hippocampus [10]. However, it is worth mentioning that 3-month-old Tg neocortex shows an opposite SOD2 pattern, with respect to the hippocampus. Tentative explanation for this behavior could refer to differences in AD-associated changes in energy metabolism and in the elicited response in the two regions [22, 37]. Notwithstanding molecular alterations, we failed to detect any morphological change at the ultrastructural level in Tg neocortical neurons at this early stage.

As a likely consequence of decreased antioxidant defences, oxidatively modified nucleic acids are already detected in 3-month-old Tg neurons, particularly in the cytoplasm. Double IF, showing partial colocalization of 8-OHG with the mitochondrial marker SOD2, demonstrates early oxidative damage to mitochondrial nucleic acids, confirming that early redox cellular alterations in A*β* pathology involve mitochondrial dysfunction. Interestingly, cortical neurons appear heterogeneous in their immunoreactivity to SOD2, showing an inverse correlation between the abundance of the antioxidant enzyme and that of the oxidative damage marker, in agreement with the neuroprotective role of SOD2 [38–41].

In relation to these alterations, we studied the nuclear receptor PPAR*α*, whose role in modulating cellular redox status and repressing inflammatory signaling pathways has long been established [42]. Growing evidence indicates neuroprotective action of PPAR*α* agonists in A*β* pathology, even though the precise molecular mechanisms underlying such effects are yet to be deciphered [19, 43–45]. Our present results, in agreement with those previously reported for the hippocampal formation [10], show that PPAR*α* is more abundant in 3-month-old Tg neocortex than in WT. This enhanced expression might represent the response to early redox unbalance. Intriguingly, the predominantly cytoplasmic immunostaining found in Tg neurons could reflect a nongenomic action, that is, tethering or squelching, leading to inhibition of inflammatory gene expression [16], albeit not excluding other still unknown functions of cytosolic PPAR*α*. On the other hand, transcriptional activity of PPAR*α* should be excluded, in view of our data on peroxisomal proteins. Indeed, expression of PMP70, Pex14, AOX, THL, and CAT, all encoded by PPRE-containing genes, is unchanged in 3-month-old Tg neocortices, compared to WT. At variance, in the hippocampal formation, PPAR*α* mostly localized to the nuclear compartment, consistent with a genomic role of the transcription factor in upregulating its target genes PMP70, AOX, and CAT [10]. These differences may reflect region-specificity in terms of cell types relative abundance (astroglial versus neuronal), neuronal subtype diversity, and metabolic features involving energy metabolism [22, 37, 46].

Concerning peroxisomes, the decrease of PMP70 observed at 6 months, in the absence of alterations to other peroxisomal markers, could indicate that metabolic changes, rather than numerical reduction, of these organelles occur during physiological brain maturation. Consistent with our previous observation in the hippocampus [10], this decrease is exacerbated in Tg neocortex, demonstrating peroxisomal dysfunction consequent to A*β*-induced ROS overproduction. This condition may lead to cytosolic accumulation of long-chain unsaturated, dicarboxylic, and methyl-branched fatty acids, preferential substrates for PMP70-mediated transport [26, 27]. Among these molecules, phytanic and pristanic acids display a cytotoxic activity on brain cells, involving mitochondrial depolarization and ROS production, as demonstrated in different neural cell types and brain regions, including the neocortex [47, 48]. However, to our knowledge, information about levels of either phytanic or pristanic acid in AD patients or animal models is presently lacking. While the observed alterations to peroxisomes may participate in the disease progression, the origin of the alterations themselves might be ascribed to the direct neurotoxic action of A*β* peptide, which indeed is known to affect peroxisomal biogenesis and function [14, 49, 50]. Noteworthy, the age of 6 months is hallmarked by intracellular A*β* accumulation in Tg neocortex, undetectable at earlier stages. These data confirm and extend previous observations on the Tg2576 mouse model [5, 6], while suggesting early involvement of peroxisomal fatty acid dysmetabolism, leading to enhanced lipid peroxidation. Accordingly, and in agreement with Praticò and collaborators [51], our data demonstrate high immunoreactivity levels to acrolein in both the nucleus and the cytoplasm of 9-month-old Tg neurons. This byproduct of lipid peroxidation is the most reactive among others, inhibiting mitochondrial activity and decreasing glutathione levels [52]. Thus, acrolein has to be considered not only a lipid peroxidation marker, but also an initiator of oxidative stress in AD.

The above-mentioned age of 9 months seems crucial in the exacerbation of A*β* pathology. Ultrastructural analyses of neocortical neurons at this age demonstrate profound alterations to the cytoplasmic compartment. These abnormalities, which were milder at 6 months, mainly involve mitochondria and Golgi complex, consistent with electron microscopic observations on human tissue [53]. More specifically, mitochondria display deranged* cristae* and vacuolization, while Golgi complex shows dilated and fragmented* cisternae*, surrounded by numerous dense bodies. These morphological findings well correlate with the progression of A*β* pathology, witnessed by our IHC results showing increase in A*β* peptides concentration and first detection of oligomers, both intra- and extracellularly. Concomitantly, Congo Red staining reveals sporadic small senile plaques. Thus, our findings, in overall agreement with other authors [5, 6, 54], indicate an earlier appearance of histopathological signs in the neocortex than in the hippocampus [10]. Extracellular accumulation of A*β* induces astroglial activation, which in our samples is indeed revealed by enhanced GFAP immunoreactivity in 9-month-old Tg neocortex. Interestingly, at the same age, we show increased levels of GPX1, suggesting an adaptive response to A*β*-mediated H_2_O_2_ overproduction, also involving astroglia [55]. In 9-month-old Tg neocortex, similarly to what was observed in the hippocampus [10], we found increased PMP70 immunoreactivity, which also could be contributed by astroglia, taking into account the abundance of peroxisomes characterizing these cells [56, 57]. Thus, considering the relevant functions of these organelles in energy metabolism, with special reference to gluconeogenesis and to both inter- and intracellular lactate shuttling [58, 59], the increase in the fatty acid transporter PMP70 could be interpreted as an adaptive response of cortical astroglial peroxisomes to glucose hypometabolism, occurring early in AD [60].

At advanced A*β* pathology stages, oxidative stress exacerbates. At 12 months, expression levels of both SOD1 and SOD2 are higher in Tg than in WT, suggesting a compensatory response against superoxide-mediated damage. Accordingly, clinical findings revealed an upregulation of antioxidant enzymes in AD progression [41]. This response appears not to be triggered in the hippocampal formation, where SODs were both downregulated in 12-month-old Tg mice, with respect to their WT counterparts [10]. It is conceivable that the greater diversity in terms of neuronal subtypes in the cortical tissue may account for these region-based differences. This interpretation is supported by IHC data, showing, particularly for SOD2, strongly immunoreactive neurons, intermingled with faintly stained ones. While superoxide anions may therefore be efficiently detoxified in Tg neocortex, H_2_O_2_ (also produced by SOD activity) is unlikely to be concomitantly removed, since GPX1 and CAT levels are not increased. The progressively aggravating oxidative stress may contribute A*β* deposition and inflammation [61], leading to distortion of cortical cytoarchitecture, with abnormal neuronal layering and deposition of large senile plaques, which are surrounded by activated astrocytes (see NeuN and GFAP IHC). Concerning the biological significance of astrogliosis in AD, a dual role has been proposed, an early, beneficial one consisting of neuroprotective/metabolic support, as we propose to occur at the age of 9 months, and a late, detrimental one leading to inhibition of neurogenesis, parenchymal scarring, and self-perpetuating inflammation [62]. Indeed, onset of inflammatory processes is thought to occur late in the pathology [63–65].

Our data on 18-month-old neocortex demonstrate decreased capacity to respond to oxidative stress, since both redox status sensors and ROS-scavenging enzymes are overall reduced in Tg. Differently, aged WT mouse brain copes with oxidative stress by upregulating PPAR*α*, PGC1*α*, and SODs. Noteworthy, our IHC results show an especially high concentration of SOD2 in activated glia surrounding amyloid plaques, whilst SOD1 is scarcely present in these cells, consistent with our previous light and electron microscopic observations [66]. The critical role of SODs in AD progression is well documented and emphasised by reports on deletion versus overexpression of the respective genes [38, 39, 67].

## 5. Conclusions

In this work we report age-related molecular changes occurring in the neocortex of the Tg2576 mouse model of AD, with a focus on oxidative stress. Our results confirm and extend previous observations from others' and our own groups, supporting the view that the chosen model is eminently suited to reveal the earliest signs of the disease, even at a preclinical stage [9, 10, 22, 29, 30]. This goal is crucial to dissect the molecular mechanisms underlying A*β* pathology onset, with the ultimate goal of developing novel therapeutic strategies to prevent, or at least to slow, disease progression.

Our present data, reporting defective antioxidant defences and cytoplasmic nucleic acid oxidative damage and altered redox status sensors in Tg neurons, starting from 3 months of age, support the hypothesis that oxidative stress and mitochondrial dysfunction are early, interrelated pathogenetic factors [35, 36, 68]. Since the primary culprit of the disease, at least in our model, remains A*β* overproduction, it will be of utmost interest to study even earlier stages, considering that congenital expression of mutated APP may lead to accumulation of toxic peptides during pre-, peri-, or early postnatal brain development. On the other hand, the opportunity this AD model offers to study pathogenetic mechanisms over a relatively long-time scale [69] allowed us to follow the gradual, though dramatic, changes of oxidative stress-related molecules in the neocortex. Overall, our data demonstrate that the ability to trigger a compensatory response is conserved until 9 months of age, while at later stages all antioxidant defences appear irreversibly lost. These results are in general agreement with the vast literature on this topic, even though time sequence of oxidative stress may differ, depending on the mouse model, gender, and brain region considered [70].

Interestingly, our present and previous [10, 22] data point not only to mitochondria, but also to peroxisomes, as organelles involved in the response to the redox unbalance characterizing the earliest phases of A*β* pathology. In fact, both organelles, which are functionally interconnected, reportedly generate, respond to, and are affected by oxidative stress mediators, in physiopathological conditions [71–73]. On the other hand, at more advanced stages of disease, peroxisomes, possibly enhancing their functions in astroglia, may play a protective role, by supporting neurons with fatty acid-derived energy fuel. This phenomenon, witnessed by the significant increase in PMP70 at 9 months, coincides with the activation of astrocytes, known to be endowed with an abundant peroxisomal population. Therefore, our study suggests that therapeutic designing against AD should consider an additional target, that is, peroxisomes. As a matter of fact, peroxisome proliferators, particularly PPAR*α* natural and synthetic agonists, have been demonstrated to control glucose, lipid, and lactate homeostasis and to exert neuroprotective actions [19, 74–76]. However, the complex heterogeneity of pathological changes occurring in AD requires that any therapeutic effort have a multitargeted approach [77]. Thus, pharmacological treatments should be directed not only to protect from oxidative damage, but also to support mitochondria and peroxisomes in their energy metabolism related functions. Also, attention should be paid to both neurons and astrocytes, for their cooperation in these pathways. It remains that the primary goal to pursue is to catch the disease at its first, subtle, and hardly recognised signs [78].
